# Orbital cellulitis with panophthalmitis and scleral necrosis – a case report

**DOI:** 10.1186/s12886-023-03193-9

**Published:** 2023-11-13

**Authors:** Aurora Rodriguez, Kamran Ahmed, Nishant Tiwari, Aparna Ramasubramanian

**Affiliations:** Phoenix Children’s Hospital, 1920 E Cambridge Ave, 85,006-1464 Phoenix, AZ United States of America

**Keywords:** Case report, Panophthalmitis, Scleral necrosis, Orbital Cellulitis, Enucleation

## Abstract

**Background:**

Orbital cellulitis is common in young children and is often secondary to coexisting sinus disease. Coexisting orbital cellulitis and panophthalmitis is a rare clinical event and usually occurs secondary to trauma or from an endogenous source.

**Case presentation:**

A febrile 2-year-old male presented with periorbital inflammation and exudative retinal detachment. Imaging showed acute sinusitis and extensive orbital cellulitis. Because of progressive scleral thinning, the patient underwent enucleation.

**Conclusion:**

We present a case of concurrent orbital cellulitis, panophthalmitis, and scleral necrosis in an immunocompetent pediatric patient. Timely intervention is important to prevent life threatening complications with the rare occurrence of coexistent orbital cellulitis and panophthalmitis.

## Background

Orbital cellulitis is more common in the pediatric population with an incidence of 1.6 per 100,000 [1]. Orbital cellulitis more commonly presents in children as a complication of sinus infection most commonly the ethmoid sinus but more than one sinus can be involved in 1/3rd of patients [1]. In developing countries, surgery and trauma are other important causes of orbital cellulitis. The severe visual and life-threatening complications of orbital cellulitis include optic neuropathy, the formation of an orbital abscess, meningoencephalitis, intracranial abscesses, venous sinus thrombosis and sepsis [1]. Panophthalmitis is a rare and serious infection of the globe that affects all the layers of the eye [2]. Coexisting orbital cellulitis and panophthalmitis is a rare clinical event with poor prognosis that must be managed as an emergency.

Collection and evaluation of the patient’s clinical information was HIPAA (Health Insurance Portability and Accountability Act) compliant. Consent was obtained from the parents to allow publication of the report. Additionally, this case report adhered to the ethical principles outlined in the Declaration of Helsinki as amended in 2013.

## Case presentation

**Fig. 1:**
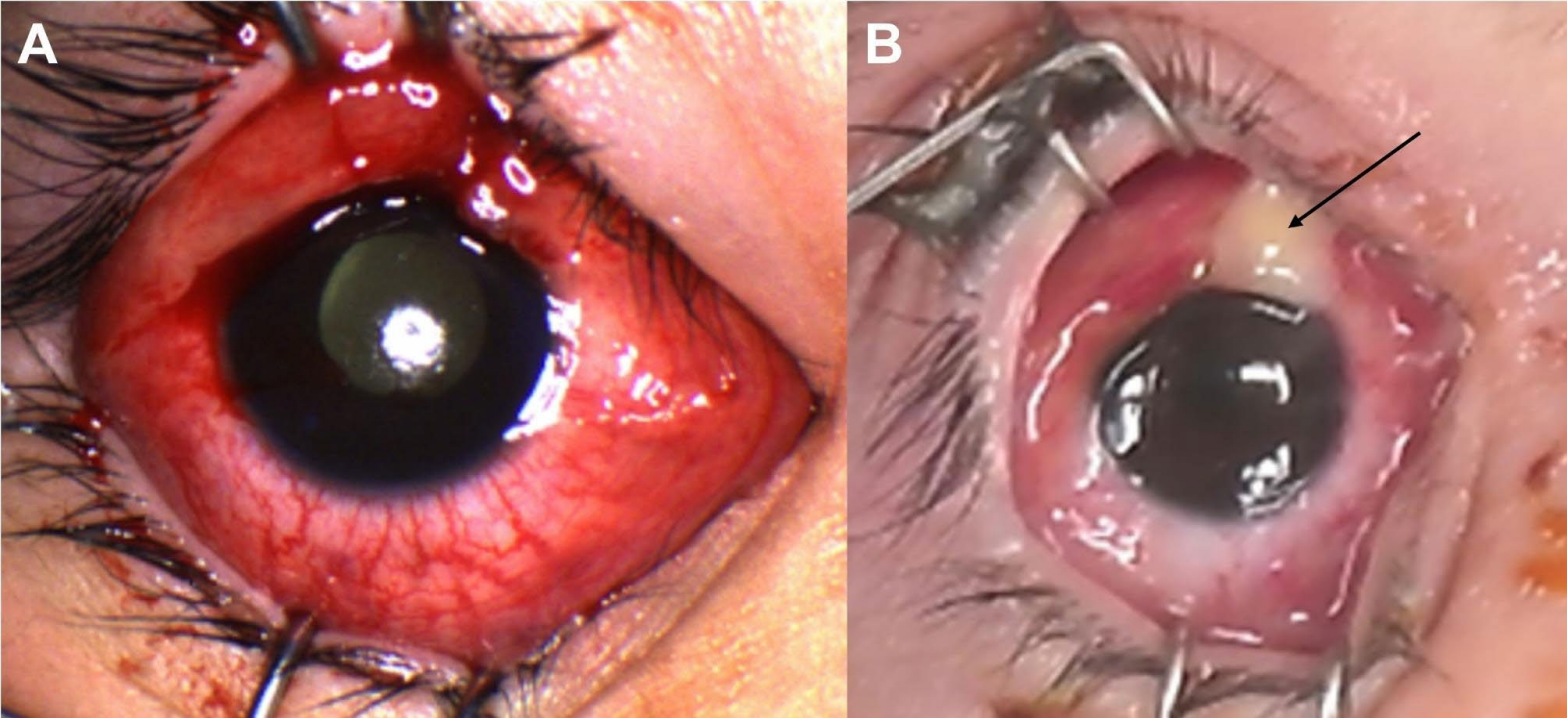
(**A**) Right periorbital inflammation and conjunctival congestion with yellow pupillary reflex at presentation. (**B**) Superior scleral thinning with purulence prior to enucleation

**Fig. 2:**
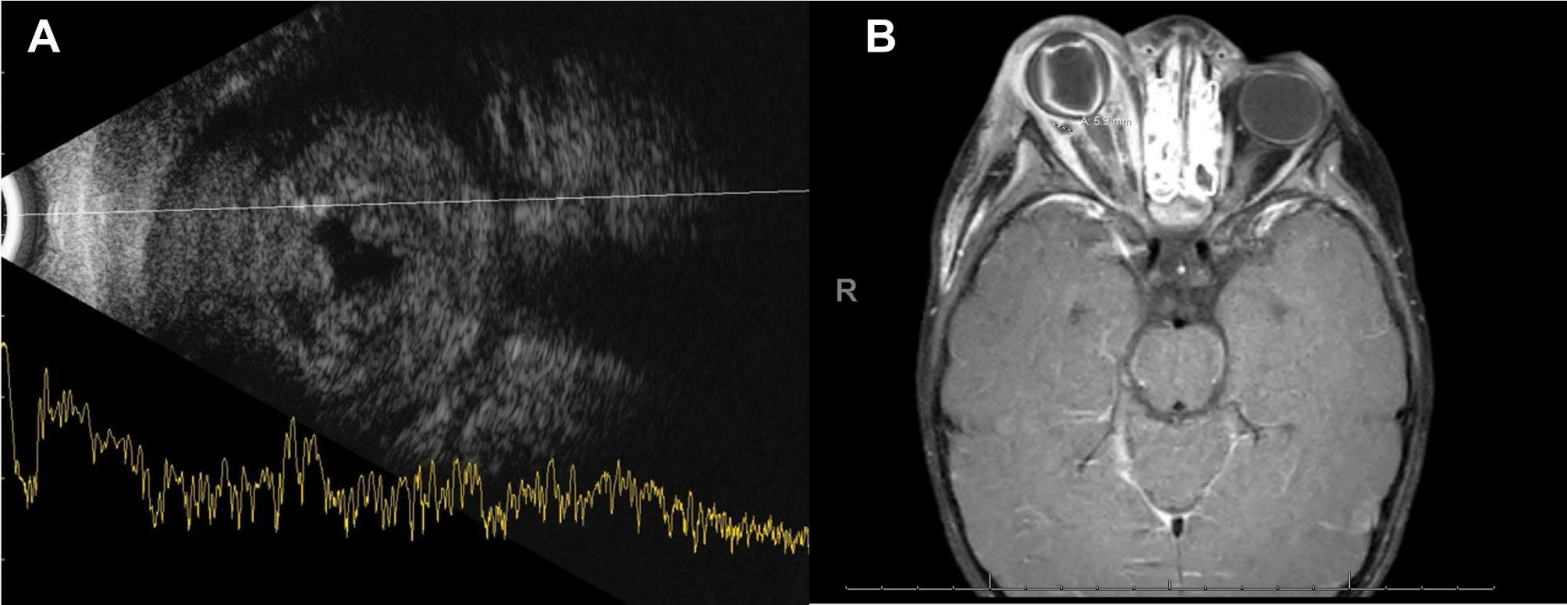
(**A**) Ocular ultrasound showing total retinal detachment with exudates. (**B**) MRI showing right globe proptosis with tenting of the posterior globe and stretched appearance of the optic nerve. There is abnormal vitreous signal with retinal detachment. Severe paranasal sinus mucosal disease was also noted

A 2-year-old male presented to the emergency department with right eye pain, swelling, discharge, and erythema (Fig. 1). He did not have a history of ocular trauma, medical comorbidities, or immunosuppression (no frequent past infections and fully immunized for age). About one week prior, the patient had developed a fever and upper respiratory symptoms for which he was treated at home with over the counter anti-pyretics. He had progressive increased eyelid edema, erythema and discharge 2 days prior to presentation. On examination, he was febrile with a temperature of 39.6 C (103.28 F) and was tachycardic (164 bpm). A respiratory PCR was positive for Influenza A virus. Ophthalmic examination revealed right periorbital edema and erythema, conjunctival congestion, and fundus examination showed retinal detachment (Fig. 1A). Ultrasound B scan showed a total retinal detachment with exudates under the retina (Fig. 2A). Facial and Orbital CT scans revealed extensive orbital cellulitis with significant inflammatory changes in the right globe, as well as extraconal areas with patchy enhancement of the right sclera. Opacification of the paranasal sinuses was also noted. The patient was promptly started on IV ceftriaxone and vancomycin and underwent right maxillary antrostomy with soft tissue removal and a right ethmoidectomy. The sinus cultures demonstrated a growth of Streptococcus mitis that was sensitive to cefepime, clindamycin, vancomycin and levofloxacin and was resistant to ceftriazone. The blood cultures were negative.

The patient had worsening symptoms and underwent MRI imaging of the brain and orbits that revealed severe paranasal sinus, and right globe proptosis with tenting of the posterior globe. Extensive inflammatory changes were also seen in the periorbital tissue (Fig. 2B). The patient developed a superior area of scleral thinning (Fig. 1B). In view of the progressive changes and scleral thinning, right eye enucleation was performed. Pathology revealed abundant necro-inflammatory debris filling the vitreous humor and extending deep to the retina into the choroid and extending peri-vascularly through the sclera into the orbital soft tissue.

The patient’s symptoms improved following enucleation, and he was continued on oral levofloxacin for 2 weeks. The patient has had no recurrent symptoms after 4 months of follow up.

## Discussion

Orbital cellulitis tends to present in children, and it is usually secondary to a sinus infection [1, 3]. Anatomical features such as a thin medial orbital wall, lack of lymphatic vessels, valveless veins of the orbit, and foramina of the orbital bones facilitate spread of infection from the sinuses to the orbit. However, even with adequate treatment, orbital cellulitis can progress quickly and lead to various complications [1, 4].

Chandler’s classification has been used to describe orbital complications of acute sinusitis and it is notes as below [1]:

Group 1: Preseptal cellulitis.

Group 2: Orbital cellulitis.

Group 3: Subperiosteal abscess.

Group 4: Intraorbital abscess.

Group 5: Cavernous sinus thrombosis (CST).

The cornerstone of management of orbital cellulitis is medical management with antibiotics based on the sensitivity of the microorganism following culture. The American Academy of Pediatrics advises empiric treatment with vancomycin ( to cover MRSA) with cefotaxime and metronidazole or clindamycin to provide concurrent coverage against gram-negative and anaerobic organisms.1 Surgical management includes drainage of orbital abscesses, sinus surgery and treatment of intracranial complications.

To the authors’ knowledge, this is the first reported case of orbital cellulitis complicated by panophthalmitis and scleral necrosis in a pediatric patient without coexisting medical comorbidities or immunosuppression. The only previous reported patients of scleral necrosis with orbital inflammation have been in pediatric patients with retinoblastoma with orbital presentation. Scleral necrosis is a severe inflammation of the sclera that has a rapid aggressive course.

Panophthalmitis is a sight- and life-threatening ocular infection that involves all tunics of the eye including the retina, choroid, and sclera with extension into the orbit. Endogenous panophthalmitis can be from hematogenous spread of the pathogen, while exogenous panophthalmitis occurs as a consequence of ocular penetration (e.g. trauma or surgery) [2]. The case reported here is unusual in that panopthalmitis resulted from direct extension from the orbit into the globe.

Patients with orbital cellulitis and panophthalmitis carry a poor ocular prognosis and should be promptly treated with broad-spectrum systemic antibiotics. Additionally, evisceration or enucleation of the globe can be done for rapidly progressing cases with no visual potential [5]. Enucleation can be especially useful in preventing and controlling life-threatening sepsis [2]. Due to the progressive nature of the disease, lack of visual potential, and the concern for other serious complications, enucleation was performed in our patient.

Our patient was immunocompetent and had a progressive disease. In a previous report of 50 immunocompromised patients with preseptal and orbital cellulitis, Sagiv et al. reported that 18% were of fungal etiology. These patients presented with less inflammatory signs and were preferably managed medically as they were poor surgical candidates [6].

This case highlights the devastating and rapidly progressive nature of coexisting panophthalmitis and orbital cellulitis. Furthermore, this case highlights the importance of early diagnosis and treatment while considering surgical management to prevent life-threatening complications.

## Data Availability

The case details are available for review if required.
